# Psoriatic Dactylitis with Onychodystrophy

**DOI:** 10.5334/jbsr.3559

**Published:** 2024-04-11

**Authors:** Lucas Van Houtven, Filip M. Vanhoenacker

**Affiliations:** 1 AZ Sint-Maarten Mechelen and University Antwerp; 2 AZ Sint-Maarten Mechelen and University Antwerp and Ghent, Liersesteenweg 435, 2800 Mechelen, Belgium

**Keywords:** Psoriasis, dactylitis, ultrasound, MRI

## Abstract

*Teaching point:* Magnetic resonance imaging (MRI) and ultrasound may be useful to assess the extent of onychodystrophy associated with psoriatic dactylitis by showing nail bed thickening, matrix changes, and vascularity indicative of active inflammation.

## Case Presentation

A 31-year-old patient with known psoriasis presented with swelling, redness, and nail dystrophy of the left great toe (Figure 1A). Conventional radiography (CR) showed subtle irregular delineated bone proliferation medially at the distal phalanx (Figure 1B, arrow) and non-specific soft tissue swelling.

**Figure 1 A:**
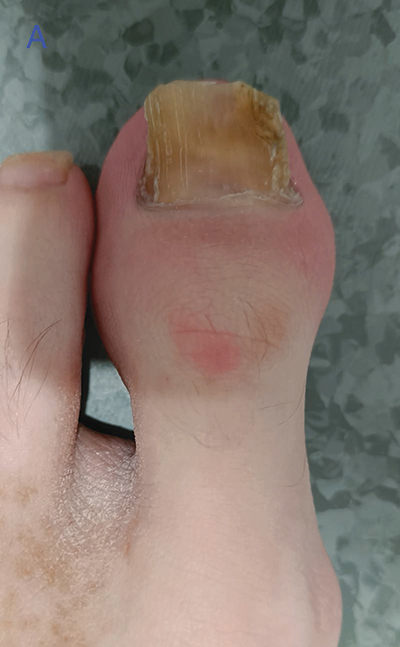
Clinical picture of the left hallux showing swelling, redness, and nail dystrophy of the left great toe.

**Figure 1 B:**
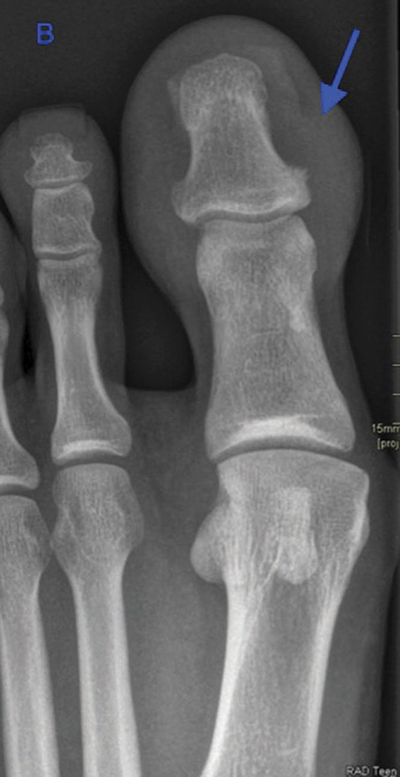
Conventional radiograph of the left foot shows non-specific soft tissue swelling and subtle irregular delineated bone proliferation medially at the distal phalanx (arrow).

Magnetic resonance imaging (MRI) revealed bone marrow edema at the distal phalanx on fat-suppressed (FS) T2-Weighted (WI) (Figure 2A, arrow). FS T1-WI after gadolinium contrast administration showed bone marrow enhancement of the distal phalanx (Figure 2B, blue arrow) and thickening and enhancement of the nail bed and underneath the nailbed (red arrow).

**Figure 2 A:**
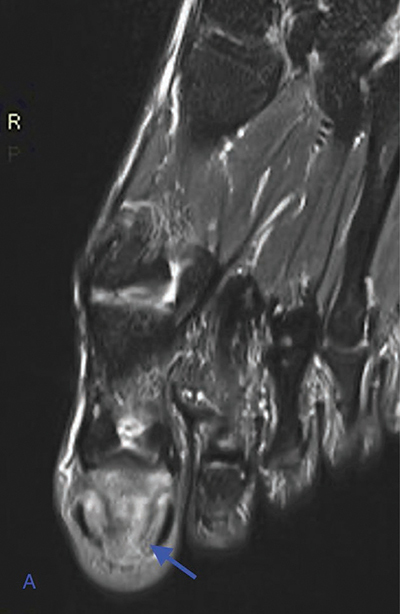
Axial FS T2-WI MRI shows bone marrow edema at the distal phalanx (arrow).

**Figure 2 B:**
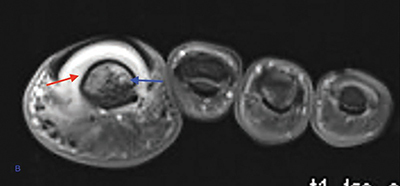
Coronal FS T1-WI after gadolinium contrast administration shows bone marrow enhancement of the distal phalanx (blue arrow) and enhancement of the nail bed (red arrow).

**Figure 3:**
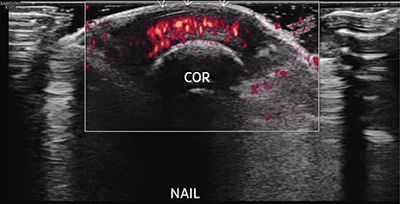
Transverse ultrasound shows increased subungeal power Doppler signal.

Subsequent ultrasound (US) confirmed increased subungual power Doppler flow signals (Figure 3, arrows), indicative of active inflammation.

Based on the combination of clinical history and imaging findings, the diagnosis of psoriatic dactylitis with onychodystrophy was made.

## Comment

Psoriatic onychodystrophy is a condition that may be associated with skin psoriasis and psoriatic arthritis (PsA). Clinically, it consists of nail abnormalities such as pitting, onycholysis, subungual hyperkeratosis, and discoloration.

The hallux is a site of predilection.

On MRI, the nail matrix and nail bed are thickened, with enhancement indicating inflammation. Fluid-sensitive sequences such as FS T2-WI and Short Tau Inversion Recovery (STIR) sequences reveal high signal within the nail bed and matrix. Bone marrow edema within the distal phalanx indicates associated psoriatic dactylitis, in keeping with more advanced disease. Diffuse swelling of the digits due to soft tissue inflammation and joint effusion, commonly referred to as “sausage digits,” can be appreciated on CR and MRI as well.

Periostitis, erosions, and bone proliferations are other well-known signs of PsA in CR [1].

US demonstrates thickening at the nail bed and underneath the nail with increased power Doppler signal, indicative of active inflammation. The US is particularly useful for monitoring the response to treatments.

Treatment for psoriatic onychopathy and dactylitis varies based on disease severity. Topical treatments suffice for mild cases, while systemic therapies are reserved for severe presentations. Early intervention is crucial to preventing disease progression.

In conclusion, the radiologist should be aware of nail abnormalities associated with psoriasis and psoriatic dactylitis. The clues to the diagnosis are the history of (cutaneous) psoriasis, typical nail abnormalities, and awareness of the imaging findings of psoriatic onychodystrophy.
